# Interleukin-1 Mediates Ischemic Brain Injury via Induction of IL-17A in γδ T Cells and CXCL1 in Astrocytes

**DOI:** 10.1007/s12017-022-08709-y

**Published:** 2022-04-06

**Authors:** Ines Sophie Schädlich, Jonas Heinrich Vienhues, Alina Jander, Marius Piepke, Tim Magnus, Kate Lykke Lambertsen, Bettina Hjelm Clausen, Mathias Gelderblom

**Affiliations:** 1 Department of Neurology, University Medical Center Hamburg-Eppendorf, Martinistrasse 52, 20246 Hamburg-Eppendorf, Germany; 2 Department of Neurobiology Research, Institute of Molecular Medicine, University of Southern Denmark, Odense, Denmark; 3 BRIDGE - Brain Research - Inter Disciplinary Guided Excellence, Department of Clinical Research, University of Southern Denmark, Odense, Denmark; 4 Deparment of Neurology, Odense University Hospital, Odense, Denmark

**Keywords:** Ischemic stroke, Inflammation, Interleukin-1, γδ T cells, Interleukin-17A, Astrocytes, CXCL1

## Abstract

As a prototypical proinflammatory cytokine, interleukin-1 (IL-1) exacerbates the early post-stroke inflammation, whereas its neutralization is protective. To further investigate the underlying cell-type-specific IL-1 effects, we subjected IL-1 (α/β) knockout (*Il1*^*−/−*^) and wildtype (WT) littermate mice to permanent middle cerebral artery occlusion (pMCAO) and assessed immune cell infiltration and cytokine production in the ischemic hemisphere by flow cytometry 24 h and 72 h after stroke. *Il1*^*−/−*^ mice showed smaller infarcts and reduced neutrophil infiltration into the ischemic brain. We identified γδ T cells and astrocytes as target cells of IL-1 signaling-mediated neutrophil recruitment. First, IL-1-induced IL-17A production in γδ T cells in vivo*,* and IL-17A enhanced the expression of the main neutrophil attracting chemokine CXCL1 by astrocytes in the presence of tumor necrosis factor (TNF) in vitro. Second, IL-1 itself was a potent activator of astrocytic CXCL1 production in vitro. By employing a novel FACS sorting strategy for the acute isolation of astrocytes from ischemic brains, we confirmed that IL-1 is pivotal for *Cxcl1* upregulation in astrocytes in vivo. Our results underscore the pleiotropic effects of IL-1 on immune and non-immune cells within the CNS to mount and amplify the post-stroke inflammatory response.

**Supplementary Information:**

The online version contains supplementary material available at 10.1007/s12017-022-08709-y.

## Introduction

Stroke ranks among the leading causes of death and disability worldwide and research over the last three decades revealed a substantial contribution of sterile inflammation to neuronal injury following ischemic stroke (Iadecola et al., 2020). In this context, interleukin-1 (IL-1) is one of the most extensively studied proinflammatory cytokines. IL-1 comprises two agonists, IL-1α and IL-1ß, which signal through the IL-1 receptor type 1 (IL-1R1) (Sims & Smith, 2010). Since the early 1990s, a magnitude of studies showed that IL-1 exacerbates ischemic brain injury in rodent stroke models (Stroemer & Rothwell, 1998; Yamasaki et al., 1995), whereas administration of the naturally occurring and highly specific IL-1 receptor antagonist (IL-1Ra) is neuroprotective (Clausen et al., 2016; Maysami et al., 2016; Relton & Rothwell, 1992). A meta-analysis of the efficacy of IL-1Ra in 25 rodent stroke studies, including a preclinical cross-laboratory study (Maysami et al., 2016), revealed a reduction in infarct volume of 36.2% after treatment (McCann et al., 2016).

In contrast to the detrimental net impact of IL-1 on acute ischemic brain damage, less is known about cell-type-specific effects of IL-1 in ischemic stroke. Among all CNS resident cells brain endothelial cells have the highest expression of IL-1R1 (Zhang et al., 2014). Its activation induces blood–brain barrier breakdown and upregulation of adhesion molecules, which lead to neutrophil transmigration (Wong et al., 2019). Consequently, brain endothelial cell-specific deletion of IL-1R1 resulted in significantly smaller infarcts after transient middle cerebral artery occlusion (tMCAO) and reduced neutrophil infiltration into the ischemic hemisphere (Wong et al., 2019).

With regard to neutrophil infiltration, IL-17A-producing γδ T cells have also been shown to promote neutrophil recruitment into the ischemic hemisphere early after stroke (Gelderblom et al., 2012); however, a possible regulation by IL-1 in the context of ischemic stroke has not been studied yet. Similar to the effects of brain endothelial IL-1R1 deletion, selective deletion of IL-1R1 on neurons significantly reduced infarct volume after tMCAO (Wong et al., 2019). Neuronal IL-1R1 deletion led to an increase in protective microglial process coverage of neurons in the penumbra, indicating that neuronal IL-1R1 signaling alters neuron-microglia interactions (Wong et al., 2019). Microglial cells, in turn, are key producers of IL-1α, IL-1ß, and IL-1Ra in the ischemic brain (Clausen et al., 2008, 2016), rather than being effector cells of IL-1 signaling.

In vitro studies showed that astrocytes are required to mediate the neurotoxic effects of IL-1ß as IL-1ß is not directly toxic to pure neuronal cell cultures (Thornton et al., 2006). Both IL-1α and IL-1ß together with other microglia-derived cytokines including TNF and complement component C1q induce astrocyte reactivity in vitro (Liddelow et al., 2017)*,* which can also be observed following experimental stroke in vivo (Zamanian et al., 2012). However, specific IL-1 effects on astrocytes following ischemic stroke have until now not been investigated in vivo.

With the present study, we aimed to extend existing knowledge on cell-type specific effects of IL-1 in ischemic stroke in vivo. Therefore, we subjected *Il1*^*−/−*^ and WT littermate mice to pMCAO and assessed immune cell infiltration and cytokine production in the ischemic hemisphere by flow cytometry 24 h and 72 h after pMCAO. We identified IL-1 as a potent activator of IL-17A production in γδ T cells in the acute phase following stroke. Moreover, we analyzed IL-1 effects on astrocytes by establishing an improved FACS sorting strategy for the acute isolation of astrocytes from ischemic hemispheres. We demonstrated that astrocytes from *Il1*^*−/−*^ mice produced significantly less neutrophil attracting C-X-C motif chemokine ligand 1 (CXCL1) when compared to their WT littermates consistent with the reduced neutrophil infiltration seen in *Il1*^*−/−*^ mice.

## Methods

### Animals

*Il1*^*−/−*^ mice, originally donated by Dr. Yoichiro Iwakura (Horai et al., 1998), and their WT littermates were maintained as a colony at the Laboratory of Biomedicine, University of Southern Denmark, Odense. Animal experiments followed the guidelines of the Danish Animal Inspectorate, and all efforts were made to minimize pain and distress (J. no. 2019-15-0201-01620).

*Tcrd-H2BeGFP* mice expressing eGFP in γδ T cells (Prinz et al., 2006) were kindly donated by Dr. Immo Prinz and maintained as a colony at the animal facility of the University Medical Center Hamburg-Eppendorf. All animal experiments performed at the University Medical Center Hamburg-Eppendorf were approved by the local animal care committee (Behörde für Lebensmittelsicherheit und Veterinärwesen Hamburg, project number N79/2019 and N59/17) and conducted according to the guidelines of the institution’s animal facility.

The number of mice required for assessing statistical significance of pre-specified effects was estimated by an a priori power analysis based on preliminary results and experience with the models used in our laboratories.

### pMCAO

Young adult male *Il1*^*−/−*^ mice, and their age-matched WT littermates were subjected to focal cerebral ischemia by pMCAO, as previously described (Clausen et al., 2008). Briefly, all animals were anesthetized by subcutaneous injections of 0.15 ml per 10 g body weight of a 1:1:2 mixture of Hypnorm™ (fentanyl citrate 0.315 mg/ml and fluanisone 10 mg/ml, VectaPharma Ltd), Midazolam (5 mg/ml, Hameln) and dH_2_O and placed on a 37 ± 0.5 °C heating pad. The distal part of the MCA was electrocoagulated via a small craniotomy made with a 0.8 mm microdrill. For post-surgical analgesia, mice were treated with Temgesic (0.001 mg/20 g buprenorphine, Reckitt Benckiser Pharmaceuticals) at a 6–8 h interval starting immediately after surgery.

### tMCAO

Homozygous young adult male *Tcrd-H2BeGFP* mice were subjected to tMCAO using the intraluminal filament method, as described previously (Arumugam et al., 2006). Briefly, mice were anesthetized with isoflurane (1.8% vol/vol oxygen) and underwent analgesia with buprenorphine (0.03 mg/kg s.c.). The left MCA was blocked for 50 min by a silicone-coated nylon filament (Docoll, 602312) introduced via the external into the internal carotid artery. Sufficient occlusion (≥ 80% reduction in regional cerebral blood flow compared to the contralateral MCA territory) was controlled by laser Doppler measurement (Moor Instruments). During surgery, a body temperature of 37 °C was maintained. For management of post-surgical pain, mice were supplied with tramadol via the drinking water.

### Grip Strength

A grip strength meter (BIO-GT-3, Bioseb) was used to measure the maximal muscle strength of both forelimbs, as previously described (Lambertsen et al., 2009). The force applied to a grid with the front paws when pulled horizontally backward by the investigator was recorded as peak tension. Individual (left/right) and total front paw grip strength was measured before (baseline) and 24 h after pMCAO. Each mouse was tested in five sequential trials, and the highest grip force (g) was recorded.

### Determination of Infarct Volume

For infarct size analysis, mice were killed 24 h after pMCAO, and brains were harvested, fresh-frozen in gaseous CO_2_, and cut into six series of 30 µm coronal cryostat sections. Toluidine blue staining and total infarct volume estimation using the Cavalieri principle were performed as described previously (Bach et al., 2012).

### Flow Cytometry

24 h or 72 h after pMCAO mice were anesthetized with isoflurane and perfused via the left ventricle with phosphate-buffered saline (PBS). Brains were harvested, and ipsilateral and contralateral hemispheres were dissected separately. Single cell suspensions were generated as previously described (Gelderblom et al., 2009). One half of the cells was immediately stained for 30 min at 4 °C with surface antibodies of the infiltration panel (Supplementary Table 1), whereas the other half was stimulated in RPMI 1640 medium (Gibco) with phorbol 12-myristate 13-acetate (100 ng/ml, Sigma-Aldrich) and ionomycin (1 µg/ml, Sigma-Aldrich) in the presence of brefeldin A (1:1000, eBioscience) for 4 h at 37 °C, 5% CO_2_ to analyze T cell cytokine production. Following stimulation, cells were stained with surface antibodies of the T cell panel (Supplementary Table 2) for 30 min at 4 °C and then fixed and permeabilized with respective buffers (Biolegend). Subsequent cytokine staining (Supplementary Table 2) was performed in permeabilization buffer for 30 min at room temperature. Afterwards, cells were washed with FACS buffer (PBS containing 0.2% w/v bovine serum albumin (BSA) (Sigma-Aldrich) and 0.5 mM EDTA (Sigma-Aldrich)) and transferred to BD TruCount™ tubes (BD Biosciences) for absolute quantification.

Cervical lymph nodes were harvested via a ventral neck incision and directly passed through a 40 µm cell strainer, which was washed with 40 ml PBS. After centrifugation, the pellet was stained as described for brain infiltrating leukocytes.

All data were acquired with a BD LSRFortessa™ flow cytometer (BD Biosciences) and analyzed with FlowJo™ version 10.8 (BD Life Sciences).

### Immunohistochemistry

Twelve or 24 h after p/tMCAO, homozygous *Il1*^*−/−*^, WT littermates and *TCRdH2eGFP* mice were anesthetized with isoflurane and perfused for 2 min with ice-cold PBS via the left ventricle followed by 20 ml of ice-cold 4% paraformaldehyde (Sigma-Aldrich). Brains were harvested and immersed in 4% paraformaldehyde at 4 °C for 24 h before being placed in a 20% or 30% sucrose solution (Sigma) until fully sunk. Before freezing the brains harvested from tMCAO mice, they were embedded in a TissueTek cryo embedding compound (Sakura). Brains were cut into 16 µm (pMCAO) or 10 µm (tMCAO) thick sections on a cryostat.

Immunofluorescent staining on tissue sections from pMCAO mice was performed as detailed in Clausen et al. (2008). As primary antibodies, mouse anti-GFAP-Cy3 (1:500, clone G-A-5, Sigma-Aldrich), rabbit anti-Iba1 (1:600, Wako), and rabbit anti-CXCL1 (1:100, Abcam) were used. AlexaFluor-488 conjugated goat anti-rabbit IgG (1:200, Invitrogen) was used to detect Iba1 and CXCL1.

On sections obtained from tMCAO mice, antigen retrieval with Proteinase K (Click-IT Plus TUNEL Assay, Invitrogen) for 15 min at room temperature and permeabilization with 0.2% Tween™ 20 (Sigma) in PBS (PBS-T) under agitation for 30 min were performed. After blocking non-specific binding sites with 1% BSA and 5% donkey serum (Sigma-Aldrich) in 0.05% PBS-T buffer, sections were stained with primary monoclonal rat anti-CD3 (1:50, clone 17A2, BD) and polyclonal chicken anti-GFAP (1:200, Millipore) overnight at 4 °C. AlexaFluor-647 conjugated goat anti-chicken IgG was used to detect GFAP (1:250, ThermoFisher), and AlexaFluor-555 conjugated donkey anti-rat IgG was used to detect CD3 (1:250, Abcam). To enhance the GFP signal, staining with polyclonal rabbit anti-GFP IgG (1:50, Invitrogen) was performed simultaneously. Finally, sections were embedded in 4’,6-diamidino-2-phenylindole (DAPI) mounting medium (Carl Roth). Representative images of the peri-infarct area were taken on a Leica SP8 confocal microscope.

### Mixed Glia and Astrocyte Culture

Primary cultures of mixed glial cells were prepared from 1 to 2 days old mice. After removal of the meninges, brains were minced in Hank’s balanced salt solution (HBSS) medium with 10 mM HEPES (Gibco), and incubated for 30 min at 37 °C in digestion solution (HBSS/10 mM HEPES with 25 U/ml Papain (Sigma) and 10 µg/ml DNase I (Roche)). After dissociation, cells were resuspended in plating media (Basal Medium Eagle (BME) supplemented with 10% fetal calf serum (FCS) and 1% penicillin/streptomycin), filtered through a 70 µm cell strainer, and plated at a density of 3 × 10^5^ cells/ml.

After 24–28 days in culture, cells were detached by trypsinization, and incubated for 20 min at 4 °C with 5 µg rat anti-CD11b (clone M1/70, Biolegend) per 10^7^ cells. After two washing steps, cells were resuspended in 1 ml BME plus 100 µl magnetic Dynabeads™ coated with polyclonal sheep anti-rat IgG (Invitrogen) which bind to CD11b^+^ microglia. After incubation for 30 min at 4 °C on a shaker, the tube was placed in a magnetic rack for 2 min. Microglia bound to the magnetic beads adhered to the walls of the tube, whereas astrocytes stayed unbound in the supernatant and were transferred to a new tube. To further increase purity, the supernatant was placed in a magnetic rack for another 2 min. Depletion of microglia and enrichment of astrocytes was controlled by flow cytometry pre- and post-Dynabeads™ treatment using a PE-conjugated antibody against GLAST (Miltenyi) and an anti-CD45 Bv421-conjugated antibody (Biolegend). Purified astrocytes were plated at a density of 2 × 10^6^ cells/well in a 6-well plate in BME supplemented with 10% FCS and 1% penicillin/streptomycin.

After 1–2 weeks, astrocytes were stimulated with TNF (10 ng/ml), IL-17A (10 ng/ml), IL-1ß (10 ng/ml), and combinations of two or all three cytokines for 24 h. Lipopolysaccharide (LPS) (1 µg/ml) was used as a positive control, medium as a negative control. After stimulation, supernatants were collected for CXCL1 quantification by ELISA, and cells were harvested for subsequent RNA isolation.

### CXCL1 ELISA

CXCL1 protein levels in cell culture supernatants were determined by ELISA as duplicates according to the manufacturer’s protocol (R&D Systems). The standard curve was created with four-parameter logistic regression.

### FACS Sorting of Astrocytes

Following transcardial perfusion with PBS, brains were harvested, and olfactory bulbs and cerebellum were removed. Ipsilateral and contralateral hemispheres were dissected separately and digested for 20 min at 37 °C in 24 U/ml Papain (Worthington) dissolved in Hibernate E (Gibco) (pH adjusted to 7.35–7.45) and another 10 min at 37 °C after addition of 1 mg/ml collagenase and 0.1 mg/ml DNase I in DMEM (Gibco). After the addition of protease inhibitor (1:25, Roche), the tissue was triturated and pressed through a 100 µm cell strainer. Cells were separated from myelin and debris by two-phase Percoll (GE Healthcare) gradient centrifugation (30% v/v and 70,5% v/v in DMEM and PBS, respectively) and washed twice with FACS buffer. Afterwards, cells were stained with surface antibodies (see Supplementary Table 3) for 30 min at 4 °C and washed twice with FACS buffer. The final pellet was resuspended in 50 µl PBS containing 0.3% v/v EDTA, and 5 µl was taken off as input material. Samples were sorted at a BD FACSAria™ Fusion Flow Cytometer (BD Biosciences) into Hibernate E medium in Eppendorf tubes coated with FCS. This protocol yielded on average 40,000 astrocytes per hemisphere, and cells from two ipsilateral or contralateral hemispheres were pooled for subsequent mRNA extraction.

### RNA Isolation and Quantitative Real-Time PCR

Mice were killed by cervical dislocation 24 h after pMCAO, and left hemispheres were homogenized in 1 ml TRIzol reagent (Ambion) using a tissue grinder and IKA® Ultra Turrax®. Next, chloroform (Sigma-Aldrich) was added, samples were centrifuged at 12,000×*g* for 15 min at 4 °C, and the upper aqueous phase was collected. RNA was precipitated by the addition of isopropyl alcohol, washed with 75% ethanol, and dissolved in Tris–EDTA buffer solution (Sigma-Aldrich). For subsequent quantitative real-time PCR (qRT-PCR), Trizol mRNA was further purified using the RNeasy Mini Kit (Qiagen) according to the manufacturer’s protocol. RNA from sorted astrocytes and respective input material was isolated using the RNeasy Micro Kit (Qiagen). RNA from cultivated astrocytes was isolated using QIA-Shredder spin columns and the RNeasy Mini Kit. RNA concentration and integrity were determined with the Agilent Bioanalyzer System.

Complementary DNA (cDNA) was transcribed using the Maxima First Strand cDNA Synthesis Kit for qRT-PCR (Thermo Fisher Scientific). Probe mixtures and qRT-PCR primers were obtained from Thermo Fisher Scientific and are listed in Supplementary Table 4. qRT-PCR analysis was performed on a LightCycler96 from Roche.

We used the geNorm algorithm implemented in qbase + software version 3.2 (Biogazelle, Zwijnaarde, Belgium) to identify the most stable reference gene for our study (Vandesompele et al., 2002). We tested eight commonly used housekeeping genes (*B2m*, *Gapdh*, Actb, *Pgk1*, *Gusb*, *Cyc1*, *Tbp,* and *Sdha*) in ten independent astrocyte samples from both control and ischemic stroke brains and identified *Sdha* as the candidate with the highest reference target stability across samples and conditions (average expression stability value: 0.25 < *M* < 0.275).

Fold changes for *Cxcl1* expression in cultivated astrocytes and purity analysis of sorted astrocytes were calculated using the ∆∆Ct method with *Sdha* as the housekeeping gene. Relative gene expression data of astrocytes from *Il1*^*−/−*^ compared to WT littermate mice were obtained with the ∆Ct method using *Sdha* as housekeeping gene and by normalizing 2^−∆Ct^ values of the control group (WT) to 1.

### Statistics

Statistical analyses were performed with the appropriate test indicated in the figure legends using GraphPad Prism version 9.2.0 for macOS (GraphPad Software, LLC). Briefly, an unpaired two-sided Student’s *t*-test was used to compare infarct volumes and relative gene expression data between *Il1*^*−/−*^ and WT littermate mice. Paired two-sided Student’s *t*-test was performed to compare grip strength values obtained from the same mouse before and after surgery. Flow cytometry data of *Il1*^*−/−*^ and WT littermate mice were analyzed with a Mann Whitney test. Repeated measures one-way ANOVA with the Geisser-Greenhouse correction and Tukey’s correction for multiple testing was performed for multiple comparisons of CXCL1 protein and mRNA levels from cultivated astrocytes. *P* ≤ 0.05 was considered statistically significant.

The data that support the findings of this study are available from the corresponding author on reasonable request.

## Results

### *Infarct Volumes and Motorsensory Deficits are Reduced in Il1*^*−/−*^* Mice*

**Fig. 1:**
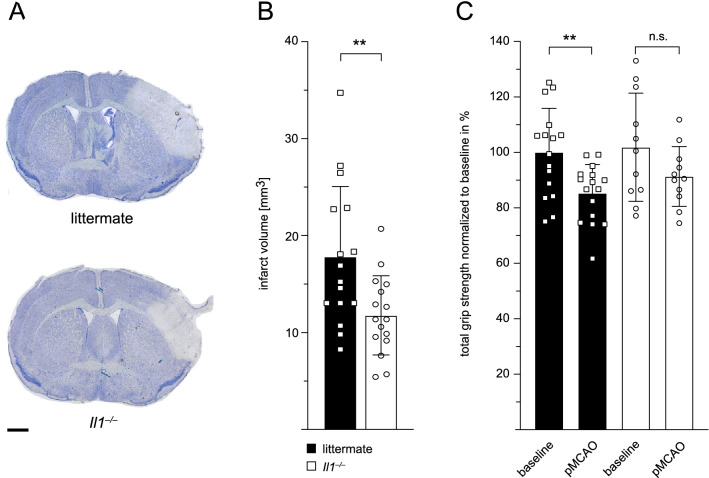
Infarct volumes and motorsensory deficits are reduced in *Il1*^*−/−*^ mice post-stroke. **A** Representative toluidine blue-stained sections for infarct volume analysis of WT littermates and *Il1*^*−/−*^ mice 24 h after pMCAO. Scale bar indicates 1 mm. **B** Infarct volumes in WT littermates and *Il1*^*−/−*^ mice 24 h after pMCAO, *n* = 16/group, unpaired Student’s *t*-test. **(C)** Total grip strength in WT littermates (*n* = 16) and *Il1*^*−/−*^ mice (*n* = 11) 24 h after pMCAO relative to their total grip strength at baseline, which was normalized to 100%, paired Student’s *t*-test. Data are presented as mean ± SD, **p ≤ 0.01

Infarct volumes were assessed 24 h after stroke induction on toluidine blue-stained sections (Fig. 1A). We detected a significant 34% reduction in infarct size in *Il1*^*−/−*^ mice when compared to WT littermates (11.78 ± 4.08 mm^3^ vs 17.81 ± 7.26 mm^3^) (Fig. 1B). The reduced infarct volume was paralleled by a reduced motorsensory deficit 24 h after pMCAO: In WT littermates, mean total grip strength post-stroke was significantly reduced by 15% on average compared to baseline measurements, whereas it was not significantly diminished in *Il1*^*−/−*^ mice, indicating that IL-1 deficiency improves functional outcome following pMCAO (Fig. 1C).

### *Neutrophil Infiltration is Reduced in Il1*^*−/−*^* Mice Post-Stroke*

**Fig. 2:**
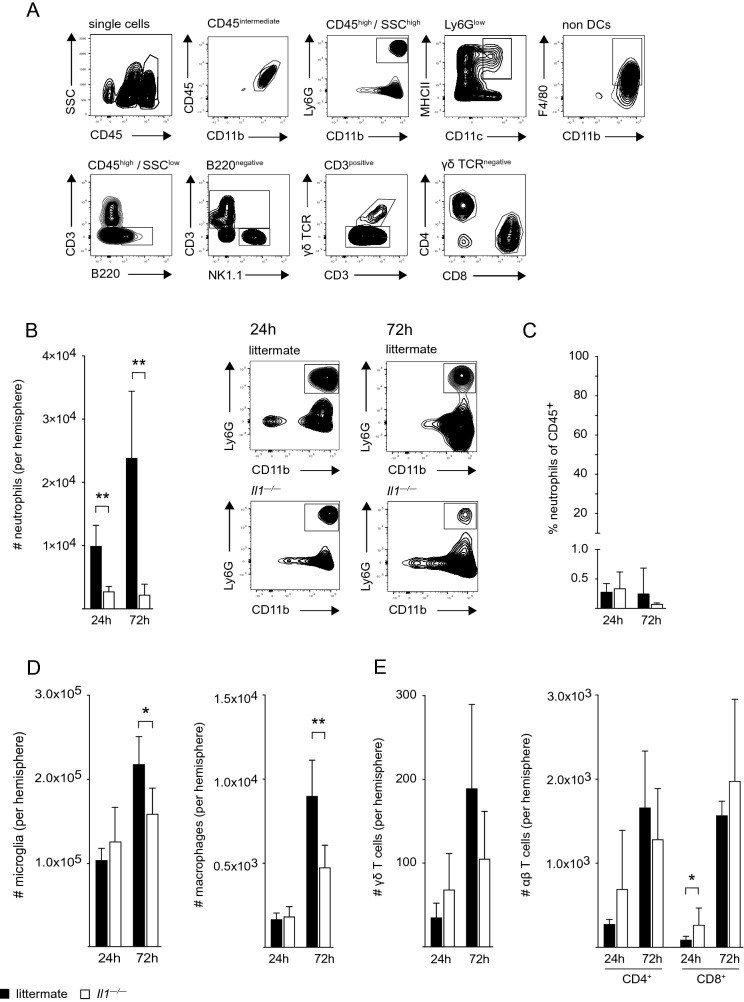
Neutrophil infiltration into the ischemic hemisphere is reduced in *Il1*^*−/−*^ mice. **A** Gating strategy: Microglia were gated as CD45^intermediate^CD11b^+^ cells. Neutrophils were gated as CD45^high^SSC^high^CD11b^+^Ly6G^+^, dendritic cells as CD45^high^SSC^high^Ly6G^low^MHCII^+^CD11c^+^ and macrophages as CD45^high^SSC^high^Ly6G^low^CD11b^+^F4/80^+^. Lymphocyte populations were gated as CD45^high^SSC^low^. B cells were excluded via B220, and NK cells were identified as CD45^high^SSC^low^CD3^−^NK1.1^+^. CD3^+^ T cells were divided into CD45^high^SSC^low^CD3^+^γδTCR^+^ γδ T cells, CD45^high^SSC^low^CD3^+^γδTCR^−^CD4^+^ CD4^+^ T cells and CD45^high^SSC^low^CD3^+^γδTCR^−^CD8^+^ CD8^+^ T cells. Respective parent populations are indicated above the plots. **B** Neutrophil cell counts (#) in the ischemic hemisphere of WT littermates and *Il1*^*−/−*^ mice 24 h and 72 h after pMCAO and representative flow cytometry plots of CD45^high^SSC^high^CD11b^+^Ly6G^+^ neutrophils. **C** Frequency of CD45^high^SSC^high^CD11b^+^Ly6G^+^ neutrophils of all CD45^+^ cells in cervical lymph nodes 24 h and 72 h after pMCAO. **D** Absolute numbers of microglia and macrophages in the ischemic hemisphere of WT littermates and *Il1*^*−/−*^ mice 24 h and 72 h after pMCAO. **E** Absolute numbers of γδ, CD4^+^ and CD8^+^ T cells in the ischemic hemisphere of WT littermates and *Il1*^*−/−*^ mice 24 h and 72 h after pMCAO. All data are presented as mean ± SD, *n* = 4–7/group, Mann Whitney test, *p ≤ 0.05, **p ≤ 0.01

Given the well-defined contribution of sterile inflammation to secondary infarct growth and neuronal injury, we next examined leukocyte infiltration into the ischemic hemisphere of *Il1*^*−/−*^ mice and WT littermate mice 24 h and 72 h after pMCAO by flow cytometry (Fig. 2A). The number of infiltrating neutrophils was significantly reduced in *Il1*^*−/−*^ mice compared to WT littermates at both time points after pMCAO (Fig. 2B). Of note, the characteristic increase in neutrophils from 24 to 72 h, when neutrophils represent the most abundant immune cell subset in WT mice (Gelderblom et al., 2009), was abolished in *Il1*^*−/−*^ mice. To account for eventual inherent differences between the two genotypes (sibling mice), we also assessed relative numbers of neutrophils in cervical lymph nodes by flow cytometry which did not differ between *Il1*^*−/−*^ mice and WT littermates (Fig. 2C). This clearly indicates that reduced neutrophil numbers in the ischemic brain of *Il1*^*−/−*^ mice are a consequence of impaired recruitment.

Regarding resident microglia and invading macrophages, we found comparable numbers in *Il1*^*−/−*^ and WT littermate mice at 24 h, but significantly lower numbers in *Il1*^*−/−*^ mice by 72 h (Fig. 2D). In the T cell compartment, we did not observe significant differences in γδ T cell or CD4^+^ T cell counts at both time points (Fig. 2E). Only numbers of CD8^+^ T cells were significantly increased in the ischemic hemisphere of *Il1*^*−/−*^ mice at 24 h.

### *IL-17A Production by γδ T Cells is Reduced in Il1*^*−/−*^* Mice*

Given that IL-17A producing γδ T cells play a crucial role in neutrophil infiltration following stroke (Gelderblom et al., 2012) and are known to express IL1-R1 (Sutton et al., 2009), we hypothesized that IL-1 also drives neutrophil infiltration into the ischemic hemisphere through the induction of IL-17A in γδ T cells. Therefore, we measured IL-17A levels in γδ T cells of *Il1*^*−/−*^ and WT littermate mice 24 h and 72 h after pMCAO by flow cytometry.

**Fig. 3:**
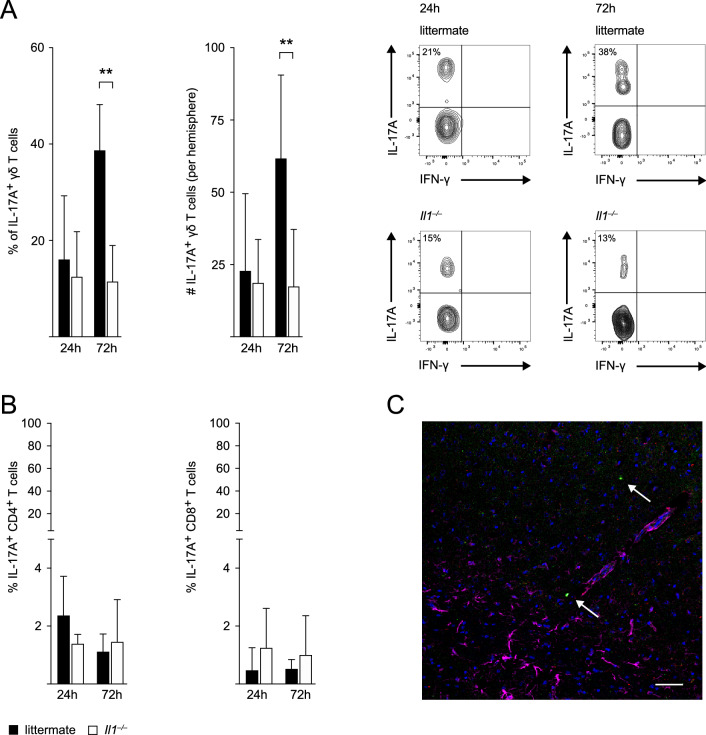
IL-17A production by γδ T cells is reduced in *Il1*^*−/−*^ mice following stroke. **A** Proportion of IL-17A producing γδ T cells of all γδ T cells and absolute numbers (#) of IL-17A positive γδ T cells in the ischemic hemisphere of WT littermates and *Il1*^*−/−*^ mice 24 h and 72 h after pMCAO (*n﻿* = 5–7/group) and representative flow cytometry plots of IL-17A^+^ γδ T cells (CD45^high^SSC^low^CD3^+^γδTCR^+^). **B** Proportion of IL-17A producing CD4^+^ and CD8^+^ T cells of all CD4^+^ and CD8^+^ T cells, respectively, in the ischemic hemisphere of WT littermates and *Il1*^*−/−*^ mice 24 h and 72 h after pMCAO (*n* = 5–7/group). **C** Immunohistochemistry of brain sections from *Tcrd-H2BeGFP* mice 24 h after tMCAO for visualization of GFP (green) and CD3 (AlexaFluor-555, red) double-positive γδ T cells (arrows) and GFAP (AlexaFluor-647, pink) positive astrocytes in the ischemic penumbra. 10 × magnification, scale bar indicates 50 µm. Data are presented as mean ± SD, Mann Whitney test, **p ≤ 0.01

Indeed, we identified γδ T cells as target cells of IL-1 signaling post-stroke. The percentage of IL-17A producing γδ T cells was significantly lower in *Il1*^*−/−*^ mice when compared to WT littermates 72 h after pMCAO (Fig. 3A). By 72 h, 38.7% of all γδ T cells were IL-17A positive in WT littermates whereas only 11.49% of all γδ T cells produced IL-17A in *Il1*^*−/−*^ mice. Absolute numbers of IL-17A^+^ γδ T cells 72 h post-stroke were also significantly reduced in the ischemic hemisphere of *Il1*^*−/−*^ mice and the typical increase in IL-17A^+^ γδ T cells from 24 to 72 h post-stroke was abrogated in *Il1*^*−/−*^ mice. In contrast to γδ T cells, less than 4% of CD4^+^ T cells and less than 2% of CD8^+^ T cells produced IL-17A in both genotypes (Fig. 3B). These results confirmed previous findings in tMCAO and validated the concept of γδ T cells as the primary source of IL-17A after pMCAO. The presence of γδ T cells in the ischemic hemisphere was additionally confirmed by immunohistochemistry of brain sections from *Tcrd-H2BeGFP* mice 24 h after tMCAO where GFP and CD3 double-positive γδ T cells were visualized in close proximity to GFAP^+^ astrocytes in the penumbra (Fig. 3C).

### *IL-1ß Induces CXCL1 Production in Astrocytes *In Vitro

**Fig. 4:**
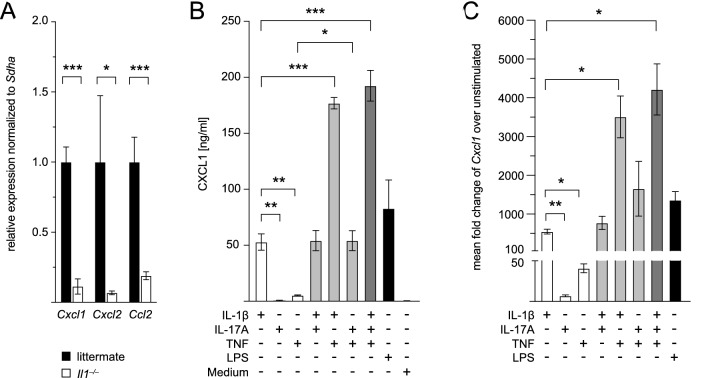
*Cxcl1* levels are reduced in the ischemic hemisphere of *Il1*^*−/−*^ mice, and astrocytes produce CXCL1 upon stimulation with IL-1ß in vitro. **A** Expression of *Cxcl1, Cxcl2,* and *Ccl2* in whole-brain mRNA from the ischemic hemisphere of *n* = 5 *Il1*^*−/−*^ mice normalized to levels in *n* = 3 WT littermate mice (normalized to 1) 24 h after pMCAO, data are presented as mean ± SEM, unpaired Student’s *t*-test. **B** CXCL1 levels in the supernatant of cultivated astrocytes 24 h after stimulation with IL-1ß, IL-17A, TNF, LPS and medium and combinations of two or all three cytokines, respectively, *n* = 4 independent experiments. Data are presented as mean ± SD, repeated measures one-way ANOVA. **C** Fold change of *Cxcl1* expression in astrocytes stimulated with IL-1ß, IL-17A, TNF, LPS and combinations of two or all three cytokines, respectively, over unstimulated astrocytes, *n* = 5 independent experiments. Data are presented as mean ± SEM, repeated measures one-way ANOVA. *p ≤ 0.05, **p ≤ 0.01, ***p ≤ 0.001

Previous in vitro experiments from our laboratory revealed that astrocytes produce the main neutrophil attracting chemokine CXCL1 upon co-stimulation with IL-17A and TNF to mediate neutrophil infiltration (Gelderblom et al., 2012). Given that astrocytes also express IL-1R1 (Zhang et al., 2014), the above findings prompted us to hypothesize that impaired neutrophil recruitment in *Il1*^*−/−*^ mice might be a consequence of reduced CXCL1 production in astrocytes. To test this hypothesis, we first assessed *Cxcl1* levels in whole-brain mRNA from ischemic hemispheres of *Il1*^*−/−*^ and WT littermate mice at 24 h by qRT-PCR. Indeed, *Cxcl1* expression was significantly reduced in *Il1*^*−/−*^ mice (Fig. 4A). The same was true for *Cxcl2* and the monocyte attracting chemokine *Ccl2*. We validated these results with mRNA isolated from FACS input single-cell suspensions of *Il1*^*−/−*^ and WT littermate mice 24 h and 72 h after pMCAO. In line with Trizol whole-brain mRNA data, *Cxcl1* and *Cxcl2* levels were profoundly ﻿reduced in *Il1*^*−/−*^ mice 24 h after stroke, whereas *Ccl2* was not significantly downregulated in input material from *Il1*^*−/−*^ mice (Supplemental Fig. 1A). Interestingly, *Cxcl1* expression was persistently reduced 72 h after stroke in *Il1*^*−/−*^ mice, whereas *Cxcl2* and *Ccl2* already tended to normalize to WT levels (Supplemental Fig. 1B).

We next stimulated primary astrocyte cultures with IL-1ß, TNF, IL-17A and respective combinations and measured both CXCL1 protein levels in the supernatant by ELISA (Fig. 4B) and *Cxcl1* mRNA levels in cell lysates by qRT-PCR (Fig. 4C). With both approaches, we observed that among all three cytokines, IL-1ß was the strongest inducer of CXCL1 production in astrocytes in vitro and that the effect of IL-1β was significantly enhanced by co-stimulation with TNF. In line with the above-mentioned previous results, IL-17A substantially amplified the TNF response, whereas IL-17A did not enhance IL-1ß effects. Given that IL-1ß is absent in *Il1*^*−/−*^ mice and that the overall IL-17A production by γδ T cells is significantly reduced, diminished *Cxcl1* expression in the ischemic hemisphere of *Il1*^*−/−*^ mice in vivo is well explained.

### *IL-1 Induces Cxcl1 Production in Astrocytes *In Vivo

**Fig. 5:**
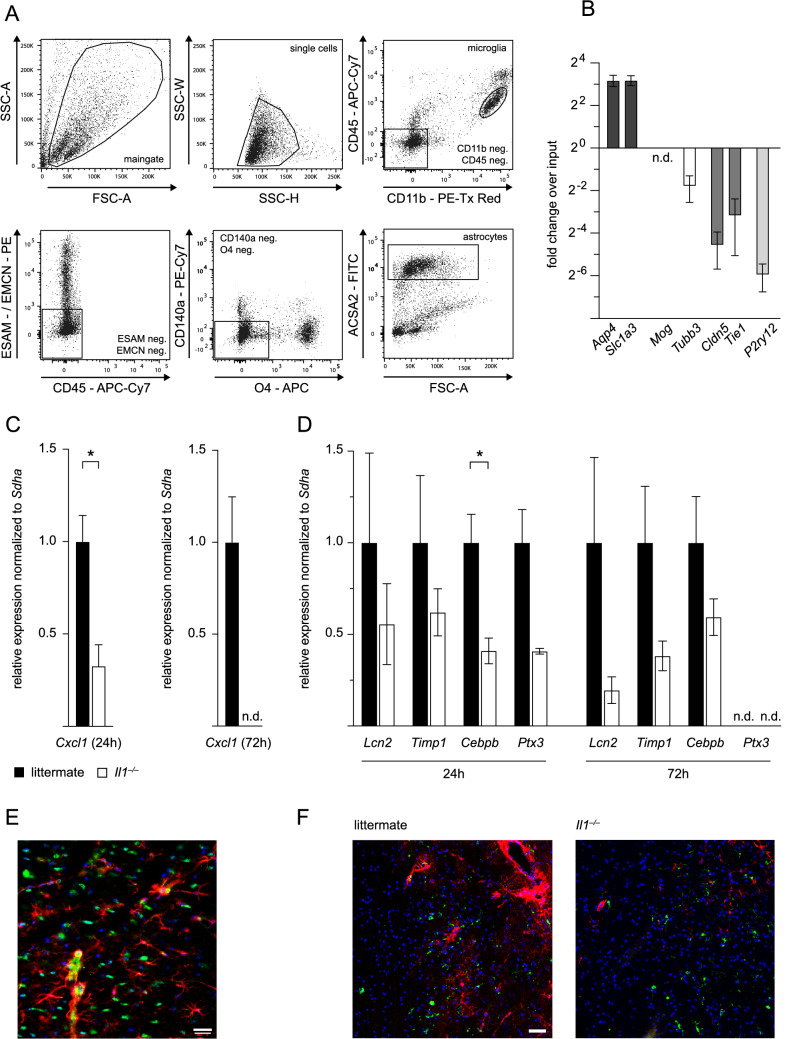
*Cxcl1* expression is reduced in astrocytes from *Il1*^*−/−*^ mice in vivo. **A** Gating strategy for FACS sorting of astrocytes. **B** Fold change over input material of astrocyte markers *Aqp4* and *Slc1a3*, oligodendrocyte marker *Mog*, neuronal marker *Tubb3*, endothelial cell markers *Cldn5* and *Tie1,* and microglia marker *P2ry12* in *n* = 8 astrocyte samples sorted from the contralateral hemisphere of ﻿ *n*= 8 *Il1*^*−/−*^ mice and *n* = 10 WT littermates at both time points after pMCAO. **C** *Cxcl1* expression 24 h and 72 h after pMCAO. *Cxcl1* expression at 24 h in *n* = 3 astrocyte samples sorted from the ischemic hemisphere of *n* = 6 *Il1*^*−/−*^ mice relative to the expression in *n *﻿ = 3 samples from *n* = 6 WT littermates, which was normalized to 1. *Cxcl1* expression at 72 h in* n * = 4 astrocyte samples sorted from the ischemic hemisphere of *n* = 7 *Il1*^*−/−*^ mice relative to the expression in *n* = 4 samples from *n* = 9 WT littermates, which was normalized to 1. **D** Expression of reactivity markers lipocalin 2 (*Lcn2*), tissue inhibitor of metalloproteinase 1 (*Timp1*), CCAAT/enhancer-binding protein beta (*Cebpb*) and pentraxin 3 (*Ptx3*) 24 h and 72 h after pMCAO in *n* = 3 astrocyte samples sorted from the ischemic hemisphere of *n* = 6 *Il1*^*−/−*^ mice relative to the expression in *n* = 3 samples from *n* = 6 WT littermates and *n* = 4 astrocyte samples sorted from the ischemic hemisphere of *n* = 7 *Il1*^*−/−*^ mice relative to the expression in *n* = 4 samples from *n*﻿ = 9 WT littermates, respectively. **E** Immunofluorescent co-staining for GFAP (Cy3, red), CXCL1 (AlexaFluor-488, green), and DAPI (blue) in WT littermate mice 12 h after pMCAO, 40 × magnification, scale bar indicates 20 µm. **F** Immunofluorescent staining for GFAP (Cy3, red), Iba1 (AlexaFluor-488, green), and DAPI (blue) in the penumbra of WT littermates and *Il1*^*−/−*^ mice 24 h after pMCAO, 40 × magnification, scale bar indicates 50 µm. All astrocyte gene expression data are presented as mean ± SEM, unpaired Student’s *t*-test, *p ≤ 0.05

As it has been demonstrated that the gene expression profile of cultivated primary astrocytes from neonatal brains significantly differs from that of adult in vivo astrocytes (Foo et al., 2011), we aimed to validate our findings on IL-1-induced astrocytic CXCL1 production in vivo. To bypass complex crossbreeding of *Il1*^*−/−*^ mice with widely applied astrocyte reporter mouse lines, we established a FACS sorting protocol for the acute isolation of astrocytes from the ischemic brain of adult mice. Previously published protocols (Mayo et al., 2014; Rothhammer et al., 2016) were refined to improve endothelial cell exclusion by selecting endothelial cell adhesion molecule (ESAM) and endomucin as two differentially expressed markers between astrocytes and brain endothelial cells (Zhang et al., 2014). Moreover, we implemented a final positive selection on ACSA-2 positive astrocytes (Batiuk et al., 2017) to further increase purity (Fig. 5A). qRT-PCR demonstrated an eight-fold enrichment of astrocyte markers *Aqp4* and *Glast/Slc1a3* in the sorted population compared to input material and a robust depletion of oligodendrocytic, neuronal, endothelial, and microglial markers (Fig. 5B).

Subsequently, we isolated astrocytes from *Il1*^*−/−*^ and WT littermate mice 24 h and 72 h after pMCAO and validated our in vitro data on astrocytic *Cxcl1* production for the first time in vivo. *Cxcl1* was significantly downregulated in astrocytes from the ischemic hemisphere of *Il1*^*−/−*^ mice relative to WT littermates 24 h after pMCAO (Fig. 5C). By 72 h, *Cxcl1* was not detectable anymore in *Il1*^*−/−*^ mice while still present in WT astrocytes. We also studied the expression of selected genes that are described to be upregulated in reactive astrocytes in response to tMCAO (Zamanian et al., 2012). We saw a consistent trend towards a lower expression of reactivity markers in astrocytes of *Il1*^*−/−*^ mice, although this regulation only reached statistical significance for one gene and timepoint (*Cebpb* at 24 h) (Fig. 5D). To confirm our data on the expression of *Cxcl1* in acutely isolated astrocytes in WT mice, we performed immunofluorescent co-stainings in brain sections from WT littermates and detected CXCL1 and GFAP double-positive astrocytes in the penumbra 12 h after pMCAO (Fig. 5E), supporting that astrocytes produce CXCL1 in vivo. Finally, we also validated the effects of IL-1 on astrocyte reactivity by immunofluorescent staining of microglial Iba1 and astrocytic GFAP on brain sections from *Il1*^*−/−*^ and WT littermate mice 24 h after pMCAO. Compared to WT controls, we saw a similar intensity of Iba1 staining in the ischemic penumbra of *Il1*^*−/−*^ mice indicative of unaffected microglia presence and activation but diminished GFAP signal pointing at reduced astrocyte reactivity (Fig. 5F).

## Discussion

In the present study, we confirmed that IL-1 deficiency is protective in a permanent model of murine stroke and leads to significantly reduced neutrophil infiltration into the ischemic hemisphere. We identified IL-1 effects on IL-17A levels in γδ T cells and *Cxcl1* expression in astrocytes as two additional mechanisms of IL-1-driven neutrophil recruitment in vivo. We argue that both mechanisms complement each other to mount and amplify the proinflammatory IL-1 response after stroke.

The protective effects of either IL-1 antagonization by IL-1RA application (Clausen et al., 2016; Maysami et al., 2016; Relton & Rothwell, 1992) or IL-1 (α/β) knockout in stroke have already been demonstrated in previous preclinical studies with IL-1 (α/β) knockout mice showing a 70% reduction in infarct volume after tMCAO (Boutin et al., 2001). In the present study, we confirmed the neuroprotective effect of IL-1 knockout for the first time in the pMCAO model, underscoring its robustness across different models of murine stroke. The dimension of infarct size reduction in *Il1*^*−/−*^ mice after pMCAO was comparable to the effects of IL-1RA treatment, which reduced infarct volume by 36.2% in a meta-analysis (McCann et al., 2016).

Consistent with previous studies on the role of IL-1 in driving neutrophil recruitment after tMCAO (Allen et al., 2012), we also demonstrated reduced neutrophil infiltration into the ischemic brain of *Il1*^*−/−*^ mice after pMCAO. IL-1 signaling in brain endothelial cells was shown to play a crucial role in mediating neutrophil recruitment after stroke since mice with brain endothelial cell-specific IL-1R1 deletion showed almost a complete loss of neutrophil infiltration following tMCAO (Wong et al., 2019) comparable to the effects seen in *Il1*^*−/−*^ mice in the present study. In these mice impaired neutrophil migration was mediated via decreased cerebrovascular expression of adhesion molecules, such as ICAM-1 and VCAM-1 (Wong et al., 2019).

In the present study, we identified IL-17A induction in γδ T cells as an additional mechanism for IL-1-mediated neutrophil recruitment in ischemic stroke. IL-1 deficiency significantly reduced the proportion of IL-17A producing γδ T cells 72 h after pMCAO. Previous work from our group demonstrated that IL-23 released by type 2 conventional dendritic cells (cDCs)-induced IL-17A production by γδ T cells and subsequent neutrophil infiltration after tMCAO (Gelderblom et al., 2018). However, IL-17A production was not completely abolished in *Il23r*^*−/−*^ mice after stroke (Gelderblom et al., 2018), which is now explained by our findings on additional IL-1 effects on IL-17A production of γδ T cells. These stimulatory effects of both IL-1 and IL-23 on IL-17A production were already demonstrated in vitro (Sutton et al., 2009), and our data clearly suggest that both pathways participate in γδ T cell activation following ischemic stroke in vivo.

As both IL-17 receptor A (IL-17RA) and IL-17RC subunits are required for signal transduction, and neutrophils lack IL-17RC expression (Sadik et al., 2011), IL-17A cannot exert direct effects on neutrophils but depends on non-immune cells to mediate neutrophil recruitment. The same holds true for IL-1, which in vitro failed to activate highly purified neutrophils in the absence of monocytes (Prince et al., 2004).

CXCL1 is considered the main neutrophil attracting chemokine in mice, and its overall expression in the ischemic hemisphere was shown to be reduced in IL-17RA knockout mice after tMCAO (Gelderblom et al., 2012). From in vitro experiments, we previously argued that astrocytes as brain resident non-immune cells mediate neutrophil attraction via CXCL1 production in response to co-stimulation with IL-17A and TNF (Gelderblom et al., 2012). In the present study, we demonstrated that stimulation with IL-1ß alone-induced CXCL1 expression in cultivated astrocytes as effectively as IL-17A and TNF together and that *Il1*^*−/−*^ mice also showed reduced *Cxcl1* levels in the ischemic brain after pMCAO compared to WT littermates. Therefore, we hypothesized that astrocytes mediate IL-1-induced neutrophil recruitment via CXCL1 expression as well and sought to validate their CXCL1 production in vivo. Acute isolation of astrocytes from the adult mouse brain has been technically challenging though essentially needed, given that astrocytes isolated from the neonatal brain have not reached their mature gene expression profile yet and exhibit substantial gene expression changes in cell culture (Foo et al., 2011). To circumvent crossbreeding of *Il1*^*−/−*^ mice with an astrocyte reporter mouse line, which is time and animal consuming, we established a FACS sorting strategy by refining previously published protocols for astrocyte isolation. With our protocol, we were indeed able to show in vivo that astrocytes express *Cxcl1* after stroke. By double-immunofluorescence staining, we pinpointed CXCL1 production to GFAP^+^ astrocytes in the peri-infarct area, thus confirming the results obtained from FACS sorted astrocytes. In line with this observation, a previous study on herpes simplex virus-1 encephalitis immunohistochemically co-localized CXCL1 and GFAP expression in perivascular astrocytes as well (Michael et al., 2020), indicating that astrocytic CXCL1 production is a common response to sterile and non-sterile neuroinflammation.

Furthermore, we demonstrated that astrocytes from *Il1*^*−/−*^ mice expressed significantly less *Cxcl1* than wildtype astrocytes. Of note, recent single-cell RNA-sequencing revealed substantial astrocyte heterogeneity under both physiological and inflammatory conditions after systemic LPS injection (Hasel et al., 2021). The fact that we still detected significant effects of IL-1 deficiency on astrocytic *Cxcl1* expression by analyzing bulk astrocytes underscores the robustness of this effect across potential astrocyte subsets with heterogeneous inflammatory responses. As a limitation, it has to be noted that IL-17A levels were also substantially reduced in *Il1*^*−/−*^ mice and that it was therefore not possible to dissect the relative contribution of IL-1 and IL-17A to astrocytic *Cxcl1* expression in vivo. Based on the cell culture experiments, we postulate that IL-1 alone is sufficient to induce *Cxcl1* expression in astrocytes and to drive subsequent amplification of the inflammatory reaction by infiltrating neutrophils. In line with this notion, functional analysis of RNA-sequencing data of astrocytes isolated from a reporter mouse line after systemic LPS injection hinted at early activation of IL-1-mediated signaling pathways in astrocytes in response to inflammatory insults (Hasel et al., 2021). Parallel activation of the IL-17A pathway via γδ T cells with comparable downstream effects probably serves to assure redundancy of inflammatory cascades as a hallmark of innate immunity.

Astrocytes undergo profound morphological, molecular, and functional changes in response to different pathological conditions in the surrounding brain tissue (Escartin et al., 2021). Analogous to other CNS injuries, extensive changes in astrocytic gene expression have also been described after tMCAO (Zamanian et al., 2012). As in vitro experiments provided evidence for a central role of at least IL-1α in the induction of astrocyte reactivity (Hasel et al., 2021; Liddelow et al., 2017), we investigated the expression of reactivity markers in acutely isolated astrocytes from the ischemic hemisphere of *Il1*^*−/−*^ and WT littermate mice 24 h and 72 h after pMCAO. Indeed, all analyzed transcripts showed a trend towards reduced expression in *Il1*^*−/−*^ mice at both early timepoints.

Lipocalin 2 (LCN2), a secretory transport glycoprotein involved in iron homeostasis, is attributed a net detrimental effect across different CNS injuries (Dekens et al., 2021). In rodent stroke, LCN2 deficiency reduced infarct volumes, and double-immunofluorescence staining identified GFAP^+^ astrocytes as the predominant cellular source of LCN2 in the penumbra 24 h after stroke (Jin et al., 2014). Comparable observations have been made for the transcription factor C/EBPbeta regulating the expression of proinflammatory genes, with C/EBPbeta null mice showing significantly smaller infarcts and lower numbers of infiltrating neutrophils three days after tMCAO (Kapadia et al., 2006). One could therefore assume that reduced expression of these two genes in astrocytes from *Il1*^*−/−*^ mice attenuates post-stroke inflammation and thus contributes to the protective phenotype of *Il1*^*−/−*^ mice. However, TIMP1 and PTX3 were also downregulated in astrocytes from *Il1*^*−/−*^ mice but were both shown to play a protective role after ischemic stroke (Rajkovic et al., 2018; Tejima et al., 2009). As we analyzed bulk astrocytes, it remains elusive whether these opposite molecular changes even occur in the same astrocyte and outweigh each other’s functional impact or whether different astrocyte subpopulations have different gene expression profiles and therefore exert counteractive functions. Despite these limitations in resolving astrocyte heterogeneity our data clearly support the notion of IL-1 as a driver of astrocyte reactivity early after stroke.

Cellular heterogeneity and the existence of complex disease- and stage-dependent astrocyte functions (Escartin et al., 2021) might also account for divergent findings on the role of reactive astrocytes in long term functional recovery after CNS injury. Of note, a recent study demonstrated, that reactive astrocytes promoted vascular repair after ischemic stroke and that ablation of reactive astrocytes impaired vascular remodeling and worsened motor recovery (Williamson et al., 2021). As our findings are limited to the acute phase after stroke up to 72 h, further studies are required to investigate the effects of IL-1 signaling on astrocytes in the context of long term regeneration and to dissect the potential contribution of different astrocyte subpopulations to functional recovery after stroke. Given that sex influences stroke outcome (Manwani et al., 2013) and aging alters the polarization of both γδ T cells (Chen et al., 2019) and astrocytes (Clarke et al., 2018) towards a proinflammatory phenotype, the exclusive use of young male mice throughout the present study is another shortcoming that should be addressed in future studies to achieve an even deeper understanding of specific IL-1 effects.

All together, we propose that IL-1 exerts its early detrimental effects in ischemic stroke via IL-17A induction in γδ T cells and that IL-1 and IL-17A signaling converge on astrocytes which in turn produce CXCL1 leading to neutrophil infiltration and subsequent amplification of the post-ischemic inflammatory reaction. This pathway complements previous reports on brain endothelial cells and neurons as target cells (Wong et al., 2019) and microglia as a key source of IL-1 in stroke (Clausen et al., 2008, 2016), underscoring the pleiotropic effects of IL-1 on different immune and non-immune cells within the CNS to mount a massive proinflammatory response upon ischemic brain injury.

## Supplementary Information

Below is the link to the electronic supplementary material.

Supplementary file1 (DOCX 180 kb)
