# Supplementary material for: Interleukin-1 Mediates Ischemic Brain Injury via Induction of IL-17A in γδ T Cells and CXCL1 in Astrocytes
Source: Neuromolecular Med. 2022 Apr 6;24(4):437–51. doi: 10.1007/s12017-022-08709-y (PMC9684245; doi:10.1007/s12017-022-08709-y)
Supplement: Supplementary file 1 — Supplementary file1 (DOCX 180 kb) [file 12017_2022_8709_MOESM1_ESM.docx]

**Supplementary Material**

Supplementary Table 1: Flow cytometry antibodies of the infiltration panel

| Antigen | Fluorochrome | Clone | Manufacturer |
| --- | --- | --- | --- |
| MHC II | FITC | M5/114.15.2 | eBioscience |
| TCRγδ | PerCP-Cy5.5 | GL-3 | Biolegend |
| Ly6G | PE | 1A8 | BD Biosciences |
| CD11b | PE-Cy7 | M 1/70 | Biolegend |
| CD11c | APC | N 418 | eBioscience |
| CD45 | APC-eFluor780 | 30-F11 | eBioscience |
| CD3 | BV 421 | 17A2 | Biolegend |
| B220 | BV 570 | RA3-6B2 | Biolegend |
| F4/80 | BV 605 | BM8 | Biolegend |
| CD8 | BV 650 | 53-6.7 | Biolegend |
| NK1.1 | BV 710 | PK 136 | Biolegend |
| CD4 | BV 785 | RM4-5 | Biolegend |

Supplementary Table 2: Flow cytometry antibodies of the T cell panel

| Antigen | Fluorochrome | Clone | Manufacturer |
| --- | --- | --- | --- |
| Interferon-g | Alexa Fluor 488 | XMG 1.2 | Biolegend |
| TCRγδ | PerCP-Cy5.5 | GL-3 | Biolegend |
| ROR-γT | PE | Q31-378 | BD Biosciences |
| IL-17 | APC | eBio17B1 | Invitrogen |
| CD45 | APC-eFluor780 | 30-F11 | eBioscience |
| CD3 | BV 421 | 17A2 | Biolegend |
| B220 | BV 570 | RA3-6B2 | Biolegend |
| CD8 | BV 650 | 53-6.7 | Biolegend |
| NK1.1 | BV 710 | PK 136 | Biolegend |
| CD4 | BV 785 | RM4-5 | Biolegend |

Supplementary Table 3: Antibodies for astrocyte sorting

| Antigen | Fluorochrome | Clone | Manufacturer |
| --- | --- | --- | --- |
| CD11b | PE-CF594 | M 1/70 | BD Horizon |
| CD45 | APC-eFluor780 | 30-F11 | eBioscience |
| ESAM | PE | 168/ESAM | Biolegend |
| EMCN | PE | eBioV.7C7 | Invitrogen |
| O4 | APC | O4 | Miltenyi |
| CD140a | PE-Cy7 | APA5 | Biolegend |
| ACSA-2 | Vio® Bright B515 (FITC) | REA969 | Miltenyi |

Supplementary Table 4: qRT-PCR primers

| Gene | Assay ID |
| --- | --- |
| *Sdha* | Mm01352366_m1 |
| *B2m* | Mm00437762_m1 |
| *Gapdh* | Mm99999915_g1 |
| *Actb* | Mm00607939_s1 |
| *Pgk1* | Mm00435617_m1 |
| *Gusb* | Mm01197698_m1 |
| *Cyc1* | Mm00470540_m1 |
| *Tbp* | Mm01277042_m1 |
| *Cxcl1* | Mm04207460_m1 |
| *Cxcl2* | Mm00436450_m1 |
| *Ccl2* | Mm00441242_m1 |
| *Aqp4* | Mm00802131_m1 |
| *Slc1a3* | Mm00600697_m1 |
| *Mog* | Mm01279062_m1 |
| *Tubb3* | Mm00727586_s1 |
| *Cldn5* | Mm00727012_s1 |
| *Tie1* | Mm00441786_m1 |
| *P2ry12* | Mm00446026_m1 |
| *Lcn2* | Mm01324470_m1 |
| *Timp1* | Mm01341361_m1 |
| *Cebpb* | Mm00843434_s1 |
| *Ptx3* | Mm00477268_m1 |


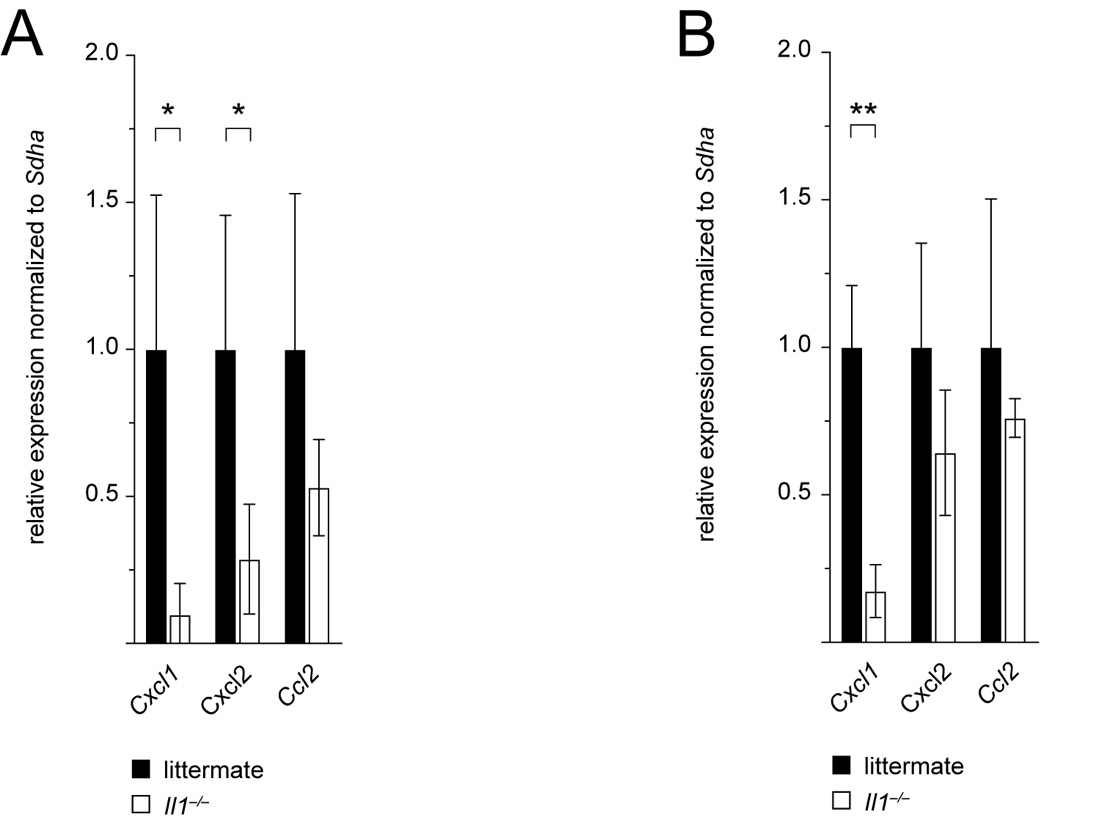


**Supplemental Figure 1:** *Cxcl1* expression is reduced 24 h and 72 h after pMCAO in *Il1^–/–^* mice.

**(A)** Expression levels of *Cxcl1*, *Cxcl2,* and *Ccl2* in FACS sorting input single-cell suspensions from the ischemic hemisphere of *Il1^–/–^* mice relative to WT littermates (normalized to 1) 24 h and **(B)** 72 h after pMCAO. Data are presented as mean ± SEM, n = 3-4 pooled samples from n = 7-8 mice/group, unpaired Student’s t-test, * p ≤ 0.05, ** p ≤ 0.01.
